# Long-Term Visual/Anatomic Outcome in Patients with Fovea-Involving Fibrovascular Pigment Epithelium Detachment Presenting Choroidal Neovascularization on Optical Coherence Tomography Angiography

**DOI:** 10.3390/jcm9061863

**Published:** 2020-06-15

**Authors:** Kyung Tae Kim, Hwanho Lee, Jin Young Kim, Suhwan Lee, Ju Byung Chae, Dong Yoon Kim

**Affiliations:** 1 Department of Ophthalmology, Gangneung Asan Hospital, University of Ulsan College of Medicine, Gangneung 25440, Korea; kkt400@gmail.com; 2 Department of Ophthalmology, Chungbuk National University Hospital, Chungbuk National University College of Medicine, Cheongju 28644, Korea; brolaril@naver.com (H.L.); cjbmed@naver.com (J.B.C.); 3 Department of Ophthalmology, Jeju National University Hospital, Jeju National University School of Medicine, Jeju 63241, Korea; umuse7@gmail.com; 4 Department of Ophthalmology, Kangwon National University Hospital, Kangwon National University Graduate School of Medicine, Chuncheon 24289, Korea; saga2407@naver.com

**Keywords:** anti-vascular endothelial growth factor, choroidal neovascularization, exudative age-related macular degeneration, fibrovascular pigment epithelial detachment

## Abstract

Background: To evaluate long-term visual/anatomic outcome after anti-vascular endothelial growth factor (anti-VEGF) therapy in patients with fovea-involving fibrovascular pigment epithelium detachment (PED) presenting with choroidal neovascularization (CNV) on optical coherence tomography angiography (OCTA). Methods: Patients with fibrovascular PED or subretinal CNV confirmed by OCTA who were treated by a relaxed treat-and-extend regimen for 2 years were retrospectively reviewed. The best-corrected visual acuity (BCVA) and central subfield retinal thickness (CST) before and after anti-VEGF injection were analyzed. Furthermore, changes in photoreceptor layer (PRL) thickness and outer retinal bands in the fovea after injection were evaluated. Results: A total of 31 eyes with fibrovascular PED and 24 eyes with subretinal CNV were included. Following a relaxed treat-and-extend regimen with anti-VEGF agents, BCVA and CST were improved, and the PRL thickness was decreased significantly. There were no differences in BCVA, CST, changes in PRL thickness, or the status of outer retinal bands between the groups. However, the difference in the amount of decrease in PRL thickness between the two groups was increased at 2 years, and the slope tended to be steeper in the subretinal CNV group. Conclusions: Exudative age-related macular degeneration (AMD) with fibrovascular PED or subretinal CNV showed good visual/anatomic outcomes after anti-VEGF treatment, regardless of the CNV type. By 2 years, fibrovascular PED did not have an additional protective effect on the outer retina, compared with subretinal CNV over 2 years. Further follow-up study might be needed to conclude that fibrovascular PED has a protective effect on the surrounding photoreceptor area.

## 1. Introduction

Age-related macular degeneration (AMD) is one of the main causes of irreversible vision loss in the elderly population in developed countries. In particular, exudative AMD-associated choroidal neovascularization (CNV) can lead to severe structural and functional damage if left untreated. However, treatment using intravitreal anti-vascular endothelial growth factor (anti-VEGF) for exudative AMD can improve visual prognosis, and many randomized clinical trials have reported the favorable efficacy of anti-VEGF for exudative AMD [1,2,3,4,5,6]. Although anti-VEGF has been proven to be effective in exudative AMD, the anatomical and functional outcomes of treatment may vary in individual patients [7,8].

Biomarkers that predict the long-term prognosis of exudative AMD have been investigated by several studies [9,10,11,12,13,14,15]. Among them, subretinal fluid (SRF) is associated with better visual functions and more benign disease courses [16,17,18,19]. A study reported that the presence of SRF could be suggestive of a functional perfused neovascular net in the foveal area, providing retinal pigment epithelium (RPE) and photoreceptor survival [19]. In addition, chronic persistent SRF, which might be associated with good visual prognosis, is mostly observed in type 1 CNV [20].

Fibrovascular pigment epithelial detachment (PED) in AMD is associated with type 1 CNV, which can be visualized by fluorescein angiography (FA) and spectral-domain optical coherence tomography (SD-OCT) [21,22]. A study reported that geographic atrophy (GA) appears to be less common in the eyes with fibrovascular PED [23]. Recently, another study also reported that macular atrophy tends to develop predominantly eccentric to the PED with long-term anti-VEGF therapy for eyes with fibrovascular PED [24]. These studies suggested a possible protective effect of fibrovascular PED on the overlying RPE and outer retina.

Considering the better visual prognosis of SRF and possible protective effect of fovea-involving fibrovascular PED on photoreceptors, the long-term outer retinal changes and visual prognosis in fovea-involving fibrovascular PED might be more favorable than in other types of CNV. Therefore, in our study, we aimed to investigate the long-term functional and anatomical prognosis and outer retinal changes in fovea-involving fibrovascular PED by comparison with fovea-involving subretinal CNV. Through these analyses, we aimed to determine if fibrovascular PED has a protective effect on the outer retina. In addition, optical coherence tomography angiography (OCTA) was used to detect CNV more accurately in this study.

## 2. Methods

A retrospective review of patients who received an intravitreal anti-VEGF injection for exudative AMD at Chungbuk National University Hospital in Korea between January 2015 and March 2018 was conducted. The main purpose of this study was to analyze the long-term visual prognosis and outer retinal changes after intravitreal anti-VEGF injections in eyes with fovea-involving fibrovascular PED (type 1 CNV) compared with eyes with fovea-involving subretinal CNV (type 2 CNV). Approval from the institutional review board and ethics committees of Chungbuk National University Hospital was obtained before the initiation of the study, which was performed in compliance with the tenets of the Declaration of Helsinki.

Inclusion criteria were patients with fovea-involving fibrovascular PED who were treated by a relaxed treat-and-extend regimen with anti-VEGF agents and who were followed up for more than 2 years. Patients with fovea-involving subretinal CNV were used as controls for comparison with fovea-involving fibrovascular PED. CNV was diagnosed and classified by FA, indocyanine green angiography (ICGA), and OCTA. Exclusion criteria included type 3 CNV, peripapillary CNV, GA or fibrovascular scar at the macula, and any history of photodynamic therapy or macular laser therapy. Other ocular conditions that could compromise visual acuity or affect image quality were also excluded. Images with a quality index less than 20 (dB) or artifacts produced by eye motion or loss of fixation were also excluded [25].

At the initial visit, all patients underwent a comprehensive ophthalmic examination, including best-corrected visual acuity (BCVA) assessment using the Snellen chart, intraocular pressure (IOP) measurement, slit-lamp examination, color fundus photography, FA, ICGA, SD-OCT (Spectralis; Heidelberg Engineering, Heidelberg, Germany) and OCTA (Carl Zeiss Meditec, Dublin, CA, USA). At each subsequent visit, patients underwent ophthalmic examinations, including assessment of the BCVA, applanation tonometry, slit-lamp examination, dilated fundus examination, fundus photography, and SD-OCT.

### 2.1. Outcome Measurement

BCVA and CST before anti-VEGF injection, at the point after the third injection, and 6 months, 1 year, and 2 years after injection were compared between the fovea-involving fibrovascular PED and subretinal CNV groups. In addition, the number of intravitreal anti-VEGF injections, choroidal thickness, and outer retinal changes including changes in the photoreceptor layer (PRL), external limiting membrane (ELM), ellipsoid zone (EZ), and cone outer segment tip (COST) line after injection for 2 years were also compared between the two groups. Patients in the fovea-involving fibrovascular PED group were divided into two groups according to the presence of SRF on SD-OCT. Eyes with persistent SRF during anti-VEGF therapy were classified into the SRF (+) group and the others were classified into the SRF (−) group. The two groups were compared to investigate whether the SRF affects visual or anatomical prognosis in fovea-involving fibrovascular PED.

### 2.2. The Relaxed Treat-and-Extend Regimen of Anti-VEGF Treatment for Exudative AMD

Initially, patients received 3 consecutive monthly intravitreal anti-VEGF injections followed by a relaxed treat-and-extend regimen allowing treatment extension by 2 weeks, dependent on disease activity (up to a maximum extension interval of 12 weeks) [26]. The disease activity during anti-VEGF treatment was defined as follows: (1) a loss of BCVA of 5 letters of more than the best BCVA recorded since baseline, (2) new retinal hemorrhage, (3) presence of fluid on SD-OCT, or (4) a combination thereof. Fluid was defined as the presence of any intraretinal fluid (resulting from disease activity as judged by the retinal specialists) and any SRF of more than 200 μm in height at the foveal center. Subfoveal SRF of 200 μm or less or any SRF elsewhere was tolerated and by itself did not prohibit extension [27].

### 2.3. SD-OCT and OCTA Analysis

**Figure 1:**
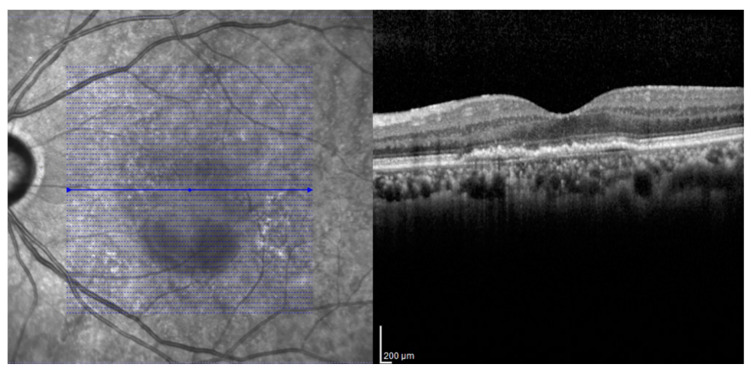
The volumetric scans of Spectralis SD-OCT. A custom 20° × 20° volume acquisition protocol which covered a 6 mm × 6 mm surface of the macula was used. With this protocol, 49 cross-sectional B-scan images were obtained, each composed of 512 A-scans.

To analyze the anatomic outcome, the central subfield thickness (CST), sub-foveal choroidal thickness (SFCT), PRL thickness, ELM, EZ, and COST line were investigated by SD-OCT. The volumetric scans of Spectralis SD-OCT were acquired with Spectralis Viewing Module (Version 6.0.9.0). A custom 20° × 20° volume acquisition protocol, which covered a 6 mm × 6 mm surface of the macula, was used to obtain one set of high-speed scans from each eye. With this protocol, 49 cross-sectional B-scan images were obtained, each composed of 512 A-scans (Figure 1). The integrated follow-up mode of the device was used to ensure that the exact same retinal area was imaged at every follow-up visit. The PRL thickness was measured as the distance from the outer margin of the outer plexiform layer (OPL) to the anterior margin of the RPE [28]. If there was SRF in the fovea, the PRL thickness was measured as the distance from the outer margin of the OPL to the outer end of the photoreceptors [29]. Segmenting of the retinal layer and measuring of the PRL thickness on SD-OCT images were manually performed by two different retinal specialists (JYK and JBC) who were masked to the study design. The average of both measurements was used for the analysis.

For OCTA images, an AngioPlex CIRRUS HD-OCT model 5000 (Carl Zeiss Meditec, Dublin, CA, USA) device was used. OCTA was performed using a 3 × 3-mm raster scan pattern centered on the fovea. The 3 × 3-mm raster scan contained 245 B-scan slices repeated up to 4 times at each B-scan position. Each B-scan comprised 245 A-scans. Images with a signal strength of less than 6 were excluded [30]. To minimize segmentation error caused by the SRF, only OCTA images with no SRF or a small amount of SRF were used for analysis. The presence of CNV on the PED was also determined by two independent observers (JYK and JBC) who were blinded to the study design and purpose. In cases of discordance in interpretation, a third observer (DYK) was asked to analyze the presence of CNV.

### 2.4. Statistical Analysis

SPSS version 22.0 software (SPSS, Inc., Chicago, IL, USA) was used for statistical analyses, and *p* < 0.05 was considered statistically significant. All values are presented as the mean ± SD or numbers (%). Normality was assessed using the Kolmogorov–Smirnov test.

Differences in parameters including BCVA, CST, and the number of injections between the fovea-involving fibrovascular PED and subretinal CNV groups were evaluated by Student’s t-test. Differences in BCVA and CST between the baseline and last visit were evaluated by the paired t-test. Pearson’s χ^2^ test was used to analyze the differences in the status of the ELM, EZ, and COST line between groups. Data in the SRF (−) and SRF (+) groups were compared by the Mann–Whitney U test and Fisher’s exact test. The reliability of the PRL thickness between the two retinal specialists was assessed by the calculating intra-class correlation coefficient (ICC).

## 3. Results

A total of 31 eyes from 31 patients with fovea-involving fibrovascular PED (aflibercept, *n* = 14; ranibizumab, *n* = 12; bevacizumab, *n* = 5) and 24 eyes from 24 patients with fovea-involving subretinal CNV (aflibercept, *n* = 9; ranibizumab, *n* = 12; bevacizumab, *n* = 3) were included in this study. The demographics and baseline ocular findings of the patients are summarized in Table 1. The mean ages of the patients were 74.6 ± 6.1 years in the fibrovascular PED group and 71.8 ± 9.1 years in the subretinal CNV group. There were no significant differences in the mean baseline BCVA (0.67 ± 0.33 logMAR in the fibrovascular PED group; 0.72 ± 0.35 logMAR in the subretinal CNV group; *p* = 0.597), the mean baseline CST (414.00 ± 70.08 μm in the fibrovascular PED group; 437.08 ± 75.08 μm in the subretinal CNV group; *p* = 0.250) or the mean baseline SFCT (231.77 ± 90.17 μm in the fibrovascular PED group; 244.42 ± 81.69 μm in the subretinal CNV group; *p* = 0.596). The number of intravitreal anti-VEGF injections was not different between the two groups at 1 and 2 years (*p* = 0.074 and *p* = 0.193, respectively).

### 3.1. BCVA Change after Anti-VEGF Treatment According to the CNV Type

**Figure 2:**
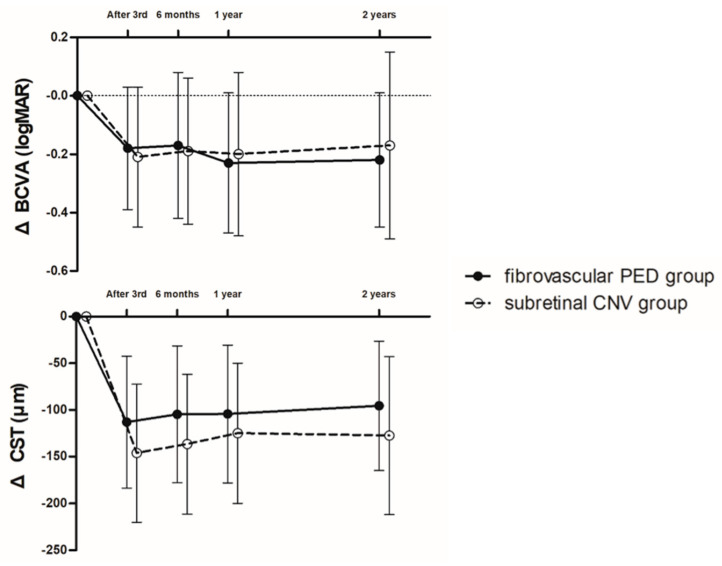
Graph illustrating changes in the amount of decrease in the logarithm of the minimum angle of resolution (logMAR), best-corrected visual acuity (BCVA), and central subfield retinal thickness (CST). The BCVA and CST at 2 years were significantly improved in the fibrovascular PED and subretinal CNV groups. However, the BCVA and CST changes from baseline at the points after the 3rd injection and 6 months, 1 year, and 2 years after intravitreal anti-vascular endothelial growth factor injection were not different between the two groups during the 2-year follow-up.

**Figure 3:**
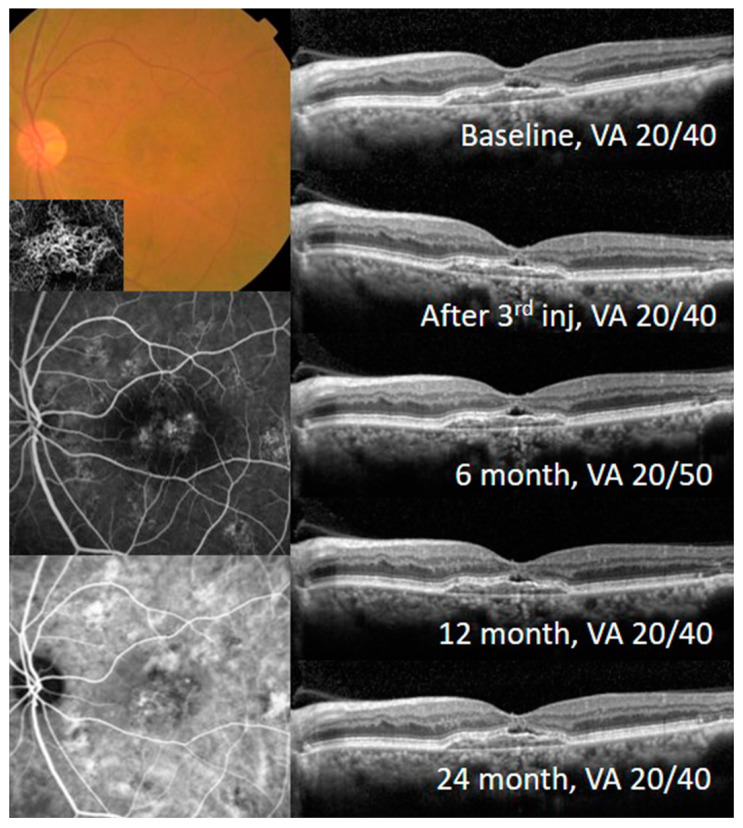
Fundus photography, fluorescein angiography (FA), indocyanine green angiography (ICGA), optical coherence tomographic angiography (OCTA) and optical coherence tomography (OCT) images of the representative case from the fibrovascular PED group. The eye of a 67-year-old male in the fibrovascular PED group was treated by a treat-and-extend regimen with aflibercept during the 2-year follow-up. Tangled choroidal neovascularization in the pigment epithelial detachment (PED) was detected by FA, ICGA, and OCTA. The subretinal fluid was observed by OCT during the follow-up period.

With the treat-and-extend treatment strategy, the BCVA and CST were significantly improved and maintained in each group. The BCVA at 2 years was improved to 0.45 ± 0.25 logMAR in the fibrovascular PED group (*p* < 0.001) and 0.55 ± 0.34 logMAR in the subretinal CNV group (*p* = 0.018). The CST at 2 years was also improved to 318.51 ± 46.13 μm in the fibrovascular PED group (*p* < 0.001) and 309.75 ± 34.08 μm in the subretinal CNV group (*p* < 0.001). The BCVA and CST changes from baseline were not different between the two groups during the 2-year follow-up (Figure 2). A representative case from the fibrovascular PED group is shown in Figure 3.

### 3.2. Photoreceptor Length Change after Anti-VEGF Treatment According to the CNV Type

**Figure 4:**
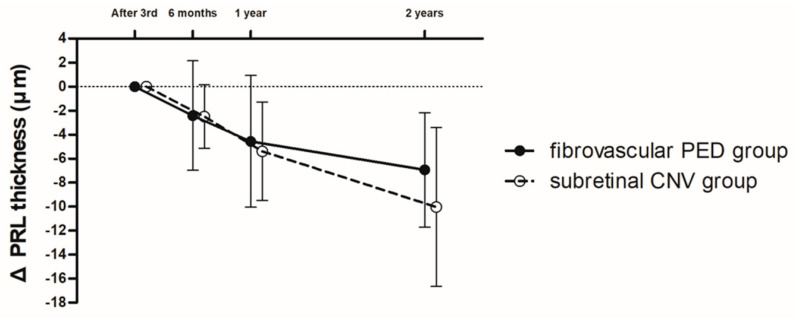
Graph illustrating changes in the amount of decrease in the photoreceptor layer (PRL) thickness. PRL thickness was continuously decreased in the fibrovascular PED and subretinal CNV groups for 2 years. However, the amount of decrease in PRL thickness was not different between the two groups during the 2-year follow-up.

In the PRL thickness, the reliability between the two retinal specialists was significantly high (ICC = 0.956, 95% CI: 0.903–0.976, *p* < 0.001). During the follow-up period, the PRL thickness was continuously decreased in the fibrovascular PED and subretinal CNV groups. The slope of the decrease in PRL thickness in the subretinal CNV group tended to be steeper than in the fibrovascular PED group; however, the amount of decrease in PRL thickness was not different between the two groups during the 2-year follow-up (Figure 4). In addition, analysis of the SFCT and outer retinal bands including the ELM, EZ, and COST line at 2 years revealed that there were no differences between the two groups (Table 2).

### 3.3. Visual Prognosis According to the Presence of Subretinal Fluid

We divided the fibrovascular PED eyes into two groups according to the presence of SRF. There were 21 eyes in the SRF (−) group and 10 eyes in the SRF (+) group. The number of intravitreal anti-VEGF injections during the follow-up was not different between the two groups. In addition, there were no significant differences in the BCVA, CST, SFCT, height of fibrovascular PED, PRL thickness, or the mean change of PRL thickness between the two groups for 2 years (Table 3).

### 3.4. Sub-Analysis of Eyes Treated with Aflibercept Monotherapy

Sub-analysis of the eyes treated with aflibercept monotherapy in the fibrovascular PED (*n* = 14) and subretinal CNV (*n* = 9) groups revealed that there were no significant differences in baseline ocular findings including the mean baseline BCVA, CST, and the number of intravitreal aflibercept injections. The BCVA at 2 years was improved to 0.50 ± 0.30 logMAR in the fibrovascular PED group (*p* < 0.001) and 0.56 ± 0.37 logMAR in the subretinal CNV group (*p* = 0.036). The CST at 2 years was also improved to 319.36 ± 37.94 μm in the fibrovascular PED group (*p* < 0.001) and 316.22 ± 35.73 μm in the subretinal CNV group (*p* = 0.001). The amount of BCVA and CST change from baseline was not different between the two groups during the 2-year follow-up. The PRL thickness was continuously decreased in the fibrovascular PED and subretinal CNV groups; however, the amount of decrease in PRL thickness was not different between the two groups during the 2-year follow-up (6.79 ± 5.07 in the fibrovascular PED group; 6.33 ± 3.24 in the subretinal CNV group; *p* = 0.815).

## 4. Discussion

In this study, we investigated long-term visual prognosis and outer retinal changes in eyes with fovea-involving OCTA-proven fibrovascular PED for patients with exudative AMD. The primary finding was that the BCVA and CST were improved and well maintained for 2 years in both the fibrovascular PED and subretinal CNV groups by the relaxed treat-and-extend strategy with intravitreal anti-VEGF injections. The secondary finding was that the PRL thickness was slightly decreased with intravitreal anti-VEGF injections, and the amount of decrease in PRL thickness was not different between the fibrovascular PED and subretinal CNV groups. Furthermore, visual outcome and outer retinal changes were not different regardless of the presence of SRF in eyes with fibrovascular PED.

Several randomized multicenter clinical trials have reported functional and anatomical improvements at 2 years in eyes with exudative AMD after receiving anti-VEGF therapy [1,2,4,5]. Although anti-VEGF treatment has been proven to be effective in exudative AMD, long-term follow-up studies have reported visual decline with chronic anti-VEGF treatment [7,8]. The mechanism that causes visual decline in eyes with long-term anti-VEGF treatment remains unclear, but several major clinical trials proposed that the development of macular atrophy or central disciform scar may play an important role in visual decline [7,8,31,32]. In addition, other studies reported that prolonged anti-VEGF treatment may induce and accelerate GA [33,34].

The visual prognosis of exudative AMD patients with large PED could be worse due to the development of RPE tears during anti-VEGF treatment. Gutfleisch et al. reported that RPE tears can be observed in 12–15% of cases as a complication after anti-VEGF treatment for PED in exudative AMD and are related to negative visual prognosis [35]. Dansingani et al. demonstrated that GA appears to occur less commonly in eyes with fibrovascular PED with prolonged anti-VEGF treatment [23]. Another study reported that macular atrophy tends to develop in a pattern predominantly eccentric to the fibrovascular PED lesion [24]. These studies indicated the protective effect of fibrovascular PED against the development of GA. Grossniklaus et al. hypothesized that fibrovascular PED recapitulates the vascular network of the choriocapillaris and provides nutrients and oxygen to an ischemic RPE or outer retina [36]. In addition, the intact RPE layer in fibrovascular PED may prevent the neovascular complex from invading the subretinal space and damaging the outer retina including the PRL [20,24]. From the point of view that fibrovascular PED might have a protective effect on the surrounding neurosensory retina, we investigated the protective effect of fibrovascular PED on the outer retina by measuring the PRL thickness and comparing it with that of eyes with subretinal CNV. In this study, we found that the outer retinal bands’ (ELM, EZ, and COST line) status after long-term anti-VEGF treatment was not different between the fibrovascular PED and subretinal CNV groups. The amounts of decrease in PRL thickness in the 2 years after intravitreal anti-VEGF treatment were also not significantly different. Therefore, fovea-involving fibrovascular PED did not have an additional protective effect on the surrounding PRL area over 2 years. However, the difference in the amount of decrease in PRL thickness between the two groups was increased at 2 years, and the slope tended to be steeper in the subretinal CNV group. Considering the steeper decrease of PRL thickness in the subretinal CNV group, the BCVA of the subretinal CNV group might be worse compared with the fibrovascular PED group, if we follow up these patients for more than two years. Therefore, further follow-up study might be needed to conclude fibrovascular PED has a protective effect on the surrounding photoreceptor area.

Studies investigating the effect of SRF on BCVA in exudative AMD treated with anti-VEGF agents have shown different results. Several previous studies found that SRF was a negative factor for BCVA in exudative AMD during anti-VEGF therapy [37,38]. On the other hand, several other studies reported that the presence of SRF was associated with a better prognosis of BCVA with anti-VEGF treatment [16,17,18]. In addition, another study reported that eyes with SRF were less likely to develop RPE atrophy even under anti-VEGF treatment [39]. A recent multicenter, randomized clinical trial also reported that eyes treated with the T&E protocol of anti-VEGF who had some SRF achieved VA that was comparable with that achieved when treatment aimed to resolve all SRF completely [27]. In this study, we analyzed the effects of SRF by comparison with the SRF (+) and SRF (−) groups in eyes with fibrovascular PED. This is the first study that analyzed the effects of SRF on visual prognosis in a specific CNV type with a relaxed treat-and-extend method. We found that there were no significant differences in BCVA, CST, PRL thickness, or mean change in PRL thickness between the two groups for 2 years with a relaxed treat-and-extend treatment method (Table 3).

Several studies suggested that the frequency of anti-VEGF injection might be associated with macular atrophy [32,34,40]. Chakravarthy et al. reported that new geographic atrophy was detected significantly more often during follow-up in participants undergoing a monthly treatment regimen than other regimens [34]. Grunwald et al. also reported that eyes receiving monthly anti-VEGF injection were at a greater risk of developing macular atrophy compared with a pro re nata (PRN) regimen [32]. These results suggest that a new injection regimen is required to reduce the number of anti-VEGF injections while showing a similar visual prognosis as monthly anti-VEGF injection regimen. A randomized, multicenter study introduced a new injection regimen, the ‘relaxed treat-and-extend regimen’, tolerating SRF in patients with exudative AMD [27]. This study demonstrated that patients treated with the relaxed treat-and-extend regimen could achieve good visual acuity with relatively fewer anti-VEGF injections. The visual outcome of these patients is comparable to the other treatment regimen, which aimed to resolve all SRF completely [27]. Therefore, we could expect that an anti-VEGF injection regimen which could minimize the number of injections while maintaining a similar visual prognosis might reduce the incidence of macular atrophy following anti-VEGF therapy in exudative AMD.

In this current study, there was no difference in the number of injections of anti-VEGF between the SRF (+) and SRF (−) group. We used a relaxed treat-and-extend treatment regimen which can allow extending the treatment interval in eyes with minimal SRF (less than 200 μm). We thought the relaxed treat-and-extend anti-VEGF regimen for AMD is the reason why the number of anti-VEGF injections was not different between SRF (+) and SRF (−) group. Considering the same visual prognosis in SRF (+) and SRF (−) fibrovascular PED patients with the treat-and-extend anti-VEGF treatment, which allows extending the treatment interval in eyes with little SRF, in the case of fibrovascular PED we know that we can extend the treatment interval even in eyes with a small amount of SRF. However, in this current study, we did not analyze the difference in visual prognosis according to the presence of SRF in subretinal CNV group. We classified eyes with persistent SRF during anti-VEGF therapy into the SRF (+) group. In the case of subretinal CNV, SRF is rarely present during treat-and-extend anti-VEGF treatment. Therefore, subgroup analysis according to the presence of SRF in subretinal CNV eyes could not be performed. Further studies might be needed to analyze the effects of long-term visual prognosis on the presence of SRF depending on the CNV type.

The reduction of choroidal thickness in eyes with exudative AMD on long-term intravitreal anti-VEGF treatment was reported in several studies [41,42,43]. Branchini et al. demonstrated that SFCT decreased from 207.4 μm to 171.8 μm in patients with exudative AMD one year after anti-VEFG treatment [41], and Ting et al. also reported that choroidal thickness decreased from 213.7 μm to 190.3 μm during 12 months of anti-VEGF treatment [43]. In addition, an animal study reported that mice lacking the soluble form of VEGF developed choroidal and RPE loss [44]. In this current study, we also found that the SFCT of eyes with fibrovascular PED or subretinal CNV was decreased from 231.7 μm to 202.1 μm, and 244.4 μm to 225.3 μm in 2 years, respectively. Based on these results, we could know that anti-VEGF therapy could cause the reduction in choroidal thickness. Furthermore, since the reduction of choroidal thickness may affect the nutritional supply to the outer retina, choroidal thinning after anti-VEGF treatment might affect the development of macular atrophy or PRL thickness decrease. Therefore, we believe that the pathophysiology of macular atrophy after anti-VEGF therapy will be better understood through further studies on the relationship between changes in SFCT and PRL thickness.

A strength of the current study is that it is the first study to analyze the protective effect of fovea-involving fibrovascular PED by a relaxed treat-and-extend regimen, compared with subretinal CNV. It was also the first study evaluating the visual/anatomic outcome of OCTA-proven CNV eyes which were a treated by a relaxed treat-and-extend regimen. However, this study has some notable limitations that were inherent in its retrospective and nonrandomized design with a small sample size. Second, as we measured the PRL thickness manually, measuring error might occur. However, we tried to minimize this error by using two independent retinal specialists who were masked to the study design to measure PRL thickness. Third, the number of anti-VEGF injections received per patient and drug type (bevacizumab, ranibizumab, and aflibercept) was not controlled; thus, a heterogeneous population was enrolled. However, considering the fact that the sub-analysis of patients treated with aflibercept alone did not differ from the overall analysis, though three different anti-VEGF injections were used, the results of this study might be valid and could reflect the actual clinical environment. Fourth, we used CST for the anatomical outcome analysis. CST that covers a relatively wider range than central foveal thickness (CFT) might be less sensitive for determining the good central visual acuity in some patients with preserved functional retina at the foveal center. However, measuring CST has the advantage of easily, accurately, and automatically identifying the change of retinal thickness in the fovea. For this reason, many large-scale clinical trials about AMD have used CST for anatomical outcome evaluation [45,46,47,48]. Furthermore, these clinical trials on AMD patients demonstrated a good correlation between CST change and BCVA change [45,46,47,48]. Therefore, we used CST for the anatomical outcome analysis. Fifth, the characterization of central visual function in the included patients might be relatively poor because objective tests for visual function, such as static perimetry or multifocal ERG, were not performed. Unfortunately, since this study was conducted by a retrospective chart review of AMD patients, we could not get data about these objective visual function tests. Using visual function tests such as static perimetry or multifocal ERG could provide the patient’s visual function more objectively; thereby, we could better know the relationship between functional visual acuity and anatomical changes in AMD patients. Therefore, further study with a relatively large number of AMD patients using objective visual function tests might be needed to confirm the current study result.

In conclusion, we found that visual prognosis was relatively good and well maintained using a treat-and-extend treatment strategy, regardless of the CNV type. In comparison with subretinal CNV, fovea-involving fibrovascular PED did not have an additional protective effect on the surrounding photoreceptor area over 2 years. However, as the slope of the decrease in PRL thickness tended to be steeper in the subretinal CNV group than in the fibrovascular PED group, further long-term follow-up research is needed to confirm a protective effect of fibrovascular PED on the outer retina. 

## Figures and Tables

**Table 1:** Patient demographics and baseline ocular findings.

Characteristics	Fibrovascular PED Group	Subretinal CNV Group	*p*-Value
No. of patients	31	24	
No. of eyes	31	24	
Age, years (mean ± SD)	74.6 ± 6.1	71.8 ± 9.1	0.194
Sex, male/female (%)	21/10 (68/22)	15/9 (63/37)	0.778
Refractive error, SE (mean ± SD)	0.32 ± 1.17	0.13 ± 1.41	0.620
Lens status, phakic/pseudophakic (%)	16/15 (52/48)	15/9 (63/37)	0.584
Best-corrected visual acuity, logMAR (mean ± SD)	0.67 ± 0.33	0.72 ± 0.35	0.597
Central subfield retinal thickness, μm (mean ± SD)	414.00 ± 70.08	437.08 ± 75.08	0.250
Subfoveal choroidal thickness, μm (mean ± SD)	231.77 ± 90.17	244.42 ± 81.69	0.596
No. of intravitreal anti-VEGF injections (mean ± SD)			
Total	11.5 ± 3.1	11.3 ± 3.1	0.795
1 year	6.7 ± 1.6	7.5 ± 1.3	0.074
2 year	4.6 ± 2.1	3.8 ± 2.2	0.193

PED = pigment epithelial detachment; CNV = choroidal neovascularization; logMAR = logarithm of the minimal angle of resolution; VEGF = vascular endothelial growth factor; SD = standard deviation.

**Table 2:** Status of outer retina and choroid at 2 years on SD-OCT after intravitreal anti-VEGF agent injections.

Characteristics	Fibrovascular PED Group	Subretinal CNV Group	*p*-Value
Status of outer retina, *n*			
ELM intact/defect	26/5	17/7	0.328
IS/OS line intact/defect	13/18	10/14	1.000
COST line intact/defect	0/31	0/24	-
Subfoveal choroidal thickness, μm (mean ± SD)	202.17 ± 92.00	225.33 ± 87.33	0.351

PED = pigment epithelial detachment; CNV = choroidal neovascularization; ELM = external limiting membrane; IS/OS = inner segment/outer segment; COST = con outer segment tip; SD = standard deviation.

**Table 3:** Clinical and OCT findings between the SRF (+) group and SRF (−) group at 2 years after anti-VEGF injections.

Characteristics	SRF (−) Group (*n* = 21)	SRF (+) Group (*n* = 10)	*p*-Value
Age, years (mean ± SD)	75.0 ± 6.9	74.0 ± 4.9	0.641
Sex, male/female (%)	14/7 (67/33)	7/3 (70/30)	1.000
No. of intravitreal anti-VEGF injections (mean ± SD)			
Total	10.9 ± 3.1	12.7 ± 2.7	0.070
1 year	6.6 ± 1.7	7.1 ± 1.3	0.342
2 year	4.3 ± 2.2	5.3 ± 1.6	0.229
Values at baseline (mean ± SD)			
BCVA, logMAR	0.70 ± 0.38	0.61 ± 0.20	0.560
CST, μm	407.48 ± 60.69	427.70 ± 88.76	0.719
SFCT, μm	228.29 ± 96.01	229.40 ± 81.98	0.882
FV-PED height, μm	151.62 ± 84.26	173.90 ± 76.26	0.320
PRL thickness, μm	108.28 ± 14.51	114.90 ± 13.91	0.124
Values at 2 years after anti-VEGF injections (mean ± SD)			
BCVA, logMAR	0.46 ± 0.28	0.43 ± 0.18	0.949
CST, μm	322.24 ± 41.26	310.70 ± 56.66	0.410
SFCT, μm	205.25 ± 97.20	196.00 ± 85.23	0.878
FV-PED height, μm	122.57 ± 28.80	136.40 ± 69.95	0.849
PRL thickness, μm	95.19 ± 11.57	102.10 ± 16.70	0.271
Δ PRL thickness from after 3rd injection	−6.81 ± 5.39	−7.20 ± 3.29	0.932

SRF = subretinal fluid; VEGF = vascular endothelial growth factor; BCVA = best-corrected visual acuity; logMAR = logarithm of the minimal angle of resolution; CST = central subfield retinal thickness; SFCT = subfoveal choroidal thickness; FV-PED = fibrovascular pigment epithelial detachment; PRL = photoreceptor layer; SD = standard deviation.

## Summary

We analyzed whether fovea-involving OCTA-proven fibrovascular PED has a protective effect on the surrounding photoreceptor area by comparison with subretinal CNV. We found that subretinal fibrovascular PED did not have an additional protective effect on the outer retina over 2 years.
